# Upcycling flavanol‐rich Chardonnay and Pinot noir grape thinned clusters as potentially functional food ingredients in cocoa‐based products

**DOI:** 10.1002/fsn3.3338

**Published:** 2023-04-04

**Authors:** Xueqi Li, Selina C. Wang

**Affiliations:** ^1^ Department of Food Science and Technology University of California‐Davis Davis California 95616 USA

**Keywords:** catechin, cocoa, epicatechin, flavanol, functional, grape marc, procyanidins, upcycling

## Abstract

In California, over 3.4 million tons of wine grapes were crushed in 2020 while every year roughly 20% of the grape mass goes unused. Grape cluster thinning at veraison, a common agricultural practice to ensure color homogeneity in wine grapes, adds to the production costs and generates substantial on‐farm loss during grapevine cultivation in which the health‐promoting values of thinned clusters (unripe grapes) are usually overlooked. In particular, the health‐promoting properties of flavanol monomers, specifically (+)‐catechin and (−)‐epicatechin, and their oligomeric procyanidins, have been extensively studied in cocoa and chocolate but not so much in grape thinned clusters in recent epidemiology studies. As part of the important agricultural by‐products upcycling effort, the current study compared thinned clusters from Chardonnay and Pinot noir, two premium wine grape varieties cultivated in California, to a traditionally Dutch (alkalized) cocoa powder that has been widely used in food applications. Thinned cluster fractions from Chardonnay and Pinot noir grapes grown in the North Coast of California showed much higher concentrations of flavanol monomers and procyanidins, with 208.8–763.5 times more (+)‐catechin, 3.4–19.4 times more (−)‐epicatechin, and 3.8–12.3 times more procyanidins (by degree of polymerization DP 1–7) than those in the traditionally Dutch cocoa powder. These flavanol‐rich thinned clusters that are also considered as plant‐based natural products suggested great potential to be functional ingredients in cocoa‐based products—which have been ubiquitously perceived as flavanol‐rich products by consumers—to enhance their overall dietary flavanol content.

## Graphical abstract

Grape thinned cluster fractions from Chardonnay and Pinot noir grapes grown in the North Coast of California showed higher concentrations of flavanol monomers and procyanidins, with 208.8–763.5 times more (+)‐catechin, 3.4–19.4 times more (−)‐epicatechin, and 3.8–12.3 times more procyanidins (by degree of polymerization DP 1–7) than a traditionally Dutch (alkalized) cocoa powder that has been widely used in food applications. Thus, grape thinned clusters could be considered as plant‐based natural products suggesting great potential to be functional ingredients in cocoa‐based products to enhance their overall dietary flavanol content.

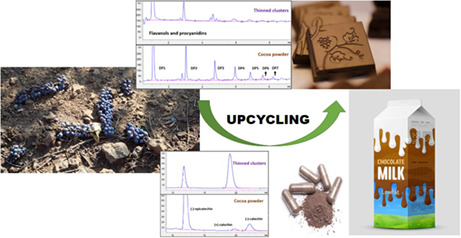

## 1 INTRODUCTION

Chardonnay and Pinot noir are two premium wine grape varieties cultivated in California. In 2020, Chardonnay (Chard) continued to be the leading white wine grape variety in California at 539,321 tons (15.2% of the total tonnage crushed), while Pinot noir (PN) was at 212,590 tons (6.0% of the total tonnage crushed) for red wine grape variety (California Department of Food and Agriculture, 2020). According to the latest California Agricultural Production Statistics, grapes (largely from the wine grape sector) ranked third among California's top‐10 valued commodities for the 2020 crop year generating $4.48 billion in cash receipts. Behind these impressive figures, the California wine industry currently suffers from a relatively high degree of waste as roughly 20% of the grape mass goes unused after winemaking (Gómez‐Brandón et al., 2019).

There are two main grape by‐products generated in the wine industry. The first is an on‐farm loss caused by cluster thinning to reduce berry variability during ripening, a practice presumed to improve wine quality. Cluster thinning at veraison is a common practice performed in California wine grape cultivation, and the timing is dependent on variety, vineyard location, and cultivation practices. The second by‐product is grape marc, which consists of grape skins, stems, seeds, and pulp accumulated either after extracting the juice to ferment white wine or after fermentation and pressing for red wine (Muhlack et al., 2018). Several studies on red and white wine grape varieties have shown that concentrations of total phenolic content, (+)‐catechin, (−)‐epicatechin, procyanidin dimers and trimers, and proanthocyanidins of wine grapes would generally decrease and then stabilize between veraison and harvests (Giovanelli & Brenna, 2007; Jordão et al., 2001a, 2001b; Kennedy et al., 2002). Thus, thinned at veraison are considered rich sources of flavanols. Nonetheless, thinned clusters are traditionally made into verjus, sour grape sauce, and unripe grape syrup in European and Middle East countries (Öncül & Karabiyikli, 2015) or simply left behind in many vineyards as soil fertilizers in spite of the increased production costs and lost yields (Preszler et al., 2010).

The health‐promoting properties of flavanol monomers, an important subgroup of the dietary polyphenols, particularly (+)‐catechin and (−)‐epicatechin, and their oligomeric procyanidins, have been extensively studied in cocoa and chocolate (Bussy, Ottaviani, & Kwik‐Uribe, 2021). Epidemiology studies have suggested that the consumption of flavanol‐rich diets and plant extracts is associated with reducing risk of cardiovascular disease (Holt et al., 2006), lowering blood pressure, especially in pre‐hypertensive and hypertensive individuals, and enhancing their physical activity (Al‐Dashti et al., 2018). Cocoa oligomeric procyanidins were found to be effective at preventing weight gain, development of glucose intolerance, and insulin resistance during a long‐term high‐fat feeding study by Dorenkott et al. (2014). Lotito and Fraga (2000) demonstrated in an in vitro study, that the addition of (+)‐catechin and (−)‐epicatechin would delay lipid oxidation, and α‐tocopherol and β‐carotene depletion in human oxidized plasma induced by a radical generator. The biological activities of (−)‐epicatechin and (−)‐epicatechin‐containing foods are of particular interests in recent years because (−)‐epicatechin causes multiple actions that may provide beneficial synergy for cardiovascular and neuropsychological health (Bernatova, 2018). It is also worth mentioning that a predominance of (−)‐catechin, rather than the more bioavailable (+)‐catechin (Ritter et al., 2010), was found in commercial chocolate samples. (+)‐Catechin is the naturally occurring form in foods such as berries and cacao beans, however, the Dutching process converts cocoa flavanols to the (−)‐catechin enantiomer (Donovan et al., 2006).

Grapes are known for their rich polyphenols including abundant (+)‐catechin, (−)‐epicatechin, and procyanidins. In an evaluation of anti‐platelet activity of grape marc extracts, the ethanolic extract with catechin, epicatechin, and quercetin being the most abundant phenolic compounds was capable of inhibiting platelets aggregation in a wide range of agonist concentrations (Choleva et al., 2019). Another recent study on the digested fractions (including flavanol monomers and dimers) of seedless red and white wine grape marc revealed that all digested fractions prevented the hyperglycemic actions in the cell viability and nitric oxide (NO)/reactive oxygen species (ROS) balance, suggesting the protective effects of grape marc products on the vascular endothelial barrier function (Gerardi et al., 2020). Holt et al. (2022) also conducted a comprehensive literature review on using chardonnay marc, a plant‐based natural product, as a new model for upcycled co‐products in food applications and comparing with considerable clinical data generated from cocoa products on human cardiometabolic health in the context of healthy dietary patterns. Thus, it is logical to explore upcycling solutions for flavanol‐rich thinned clusters in food applications, especially in cocoa‐based products that have been perceived as flavanol‐rich by consumers.

The upcycling of wine‐making by‐products in food applications has been generating more interest in recent years with the main focus on using dried grape marc as a bulking agent to change textural and rheological properties in foodstuffs such as cookies (Kuchtová et al., 2018), soft candies (Altınok et al., 2020), and chocolate spread (Acan et al., 2021). In a chocolate ice cream feasibility study conducted by Soukoulis and Tzia (2018), grape molasses (derived from grape must) was used as a potential sucrose substitution that exhibited the best chocolate color and mouth‐coating ability in the final product. Bolenz and Glöde (2021) evaluated the processing aspects and the impact on chocolate properties like particle size, total phenolic content, and sensory perception of milk chocolate enriched with grape marc and grape seed flour. However, concentration data on bioactive flavanols such as (−)‐epicatechin are beyond the study scope. When cocoa‐based products are analyzed for their flavanol content, individual ingredients, such as thinned clusters in various forms (e.g., powder, paste, etc.), are generally not separated from the matrix to gain insights on the individual contribution from each ingredient. Our study utilized improved ultra‐performance liquid chromatography‐fluorescence detector (UPLC‐FLD) methods that provided new reference information on health‐promoting flavanol monomers (+)‐catechin and (−)‐epicatechin, and procyanidins (by degree of polymerization DP 1–7) of thinned clusters alongside cocoa flavanols. The idea of incorporating grape thinned grape clusters into cocoa‐based food products as functional ingredients opens new doors to upcycle underutilized grapevine by‐products and to improve cocoa‐based products with potentially more health‐promoting and positive sensory properties with fewer calories.

## 2 MATERIALS AND METHODS

### 2.1 Chemicals and reagents

HPLC grade water and solvents methanol, acetone, hexane, acetonitrile, ethanol, and glacial acetic acid; and Folin–Ciocalteu reagent, sodium carbonate anhydrous, and ammonium acetate were purchased from Thermo Fisher Scientific. Analytical standards of gallic acid, (+)‐catechin, (−)‐catechin, and (−)‐epicatechin were obtained from MilliporeSigma. (+)‐epicatechin was purchased from Nacalai USA, Inc. The Reference Material (RM) 8403 cocoa flavanol extract was obtained from the National Institute of Standards and Technology (NIST).

### 2.2 Thinned grape cluster samples

Fresh Chardonnay and Pinot noir thinned clusters were collected at veraison from the Los Carneros American Viticultural Areas (AVA) in Sonoma County (California, USA) in mid‐August 2019. The clusters were then categorized into light and dark for Chardonnay, and red and green for Pinot noir, respectively, based on berry colors. To gain more in‐depth understanding of the flavanol composition of the thinned clusters, the seed and seedless (pulp and skin) fractions were manually separated, freeze‐dried, and milled as described in Sinrod et al. (2021).

### 2.3 Cocoa powder samples

Commercially available cocoa flavanols references, Acticoa™ (natural cocoa powder with European Union approved health claim) and Mullica™ (traditionally Dutched/alkalized cocoa powder), were obtained from Barry Callebaut USA LLC.

### 2.4 Total phenolic content

The total phenolic content of grape thinned grape clusters was measured as described by Škerget et al. (2005) with modifications. To 0.5 mL of the diluted extract (0.01 g freeze‐dried sample in 5 mL 50% ethanol: water) in a 10 mL volumetric flask, 2.5 mL of Folin–Ciocalteu reagent (diluted 1:10 with water) and 2 mL of sodium carbonate (75 g/L) were added, and filled up to the 10 mL line with DI water. The sample was incubated for 2 h in the dark before absorbance was measured at 760 nm on a Genesys 10S UV–Vis Spectrophotometer (Thermo Fisher Scientific). A calibration curve was prepared using gallic acid.

### 2.5 Flavanols and procyanidins (DP 1–7)

Flavanols and procyanidins (DP 1–7) of grape thinned grape clusters and cocoa powders were analyzed and reported following a single‐laboratory validated UPLC‐FLD method by Bussy, Hewitt, et al. (2021) and Bussy, Ottaviani, and Kwik‐Uribe (2021) with modifications. Cocoa powders were defatted three times by sonicating 5 g powder in ~45 mL hexane at 50°C for 5 min each time and completely dried in a fumehood prior to extraction. Defatting was skipped for grape thinned clusters due to low‐fat content (<10%) of seedless samples and concerns of phenolic degradation during extensive hot hexane extraction for seed fractions. For subsequent extraction of flavanols and procyanidins, 0.05 g defatted Acticoa™ cocoa powder, 0.2 g defatted Mullica™ cocoa powder, and 0.2 (seed) to 0.25 g (seedless) thinned clusters were dissolved in 5–10 mL acetone:water:acetic acid (AWAA; 7:3:0.1, by vol.), sonicated at 50°C for 5 min, and centrifuged at 5000 rcf for 10 min, respectively. Supernatants were further cleaned on Oasis PRiME MCX cartridges (Waters Corporation), diluted, and filtered through PTFE filters (0.45 μm) for UHPLC‐FLD analysis.

A Waters™ Torus diol column (100 × 3.0 mm × 1.7 μm) kept at 50°C was used for separation on an Agilent 1290 Infinity UPLC coupled with a 1260 Infinity FLD. Mobile phase A was acetonitrile: acetic acid (98:2, by vol.) and mobile phase B was methanol:water:acetic acid (95:3:2, by vol.). The gradient was following 0% B for 0.37 min to 45% B in 10.03 min to 95% B in 0.25 min and held for 2.35 min. The flow rate was 1 mL min^−1^ with a sample injection volume of 2 μL. The FLD condition was optimized at a photomultiplier tube (PMT) gain at 11 with an excitation wavelength of 230 nm and an emission wavelength of 321 nm. The quantification was determined using NIST RM 8403 as external standards.

### 2.6 Catechin and epicatechin enantiomers

Flavanol enantiomers (+)‐ and (−)‐epicatechin as well as (+)‐ and (−)‐catechin were characterized using a modified method from Machonis et al. (2014) For cocoa powders, 0.05 and 0.1 g of defatted Acticoa™ and Mullica™ samples were dissolved in 5 mL methanol:water:acetic acid (MWAA; 7:2.95:0.05, by vol.), respectively, while for grape thinned clusters, 0.1 (seed) to 0.15 g (seedless) freeze‐dried samples were dissolved in 5 mL MWAA. All mixtures underwent sonication at 50°C for 5 min and centrifuged at 5000 rcf for 10 min to obtain supernatants. Supernatants were further diluted and filtered for UPLC‐FLD analysis.

An Astec® Cyclobond® I2000 RSP (250 × 4.6 mm × 5 μm) kept at 35°C was used for separation on an Agilent 1290 Infinity UPLC‐FLD. The isocratic mobile phase was 20 mM ammonium acetate buffer (pH 4.0):methanol (7:3, by vol.). The flow rate was 1 mL min^−1^ for a total run time of 30 min with a sample injection volume of 10 μL. The FLD was set at an excitation wavelength of 276 nm and an emission wavelength of 316 nm. Five‐point calibration curves of each enantiomer were plotted for quantification.

### 2.7 Moisture content

The moisture content of freeze‐dried thinned grape clusters was determined according to Thakur et al. (2010) for reporting TPC, and flavanols and procyanidins (DP 1–7) of thinned clusters in DW.

### 2.8 Statistical analysis

The total phenolic content was measured in triplicate for thinned cluster fractions. Flavanol monomers (including catechin and epicatechin enantiomers) and procyanidins (DP 1–7) were performed in duplicate. All data are expressed as mean ± standard deviation (SD). One‐way analysis of variance (ANOVA) followed by Tukey & Dunnett multiple comparisons were applied using XLSTAT (Addinsoft Inc.) to evaluate significant differences among thinned clusters and cocoa powders flavanol content. For all comparisons, differences were considered statistically significant at a value of *p* < .05.

## 3 RESULTS AND DISCUSSION

The two wine grape varieties, Chardonnay and Pinot noir, were categorized into four different thinned cluster fractions based on berry color. Each variety that was analyzed in the current study included: Chardonnay seed and seedless dark (Chard_S_Dark and Chard_SL_Dark), Chardonnay seed and seedless light (Chard_S_Light and Chard_SL_Light), Pinot noir seed and seedless red (PN_S_Red and PN_SL_Red), and Pinot noir seed and seedless green (PN_S_Green and PN_SL_Green). To help illustrate the potential of using Chardonnay and Pinot noir grape thinned clusters as functional ingredients in cocoa‐based products, two commercial cocoa powder samples were also analyzed and used as reference samples for their monomeric and oligomeric flavanols and procyanidins content.

**FIGURE 1:**
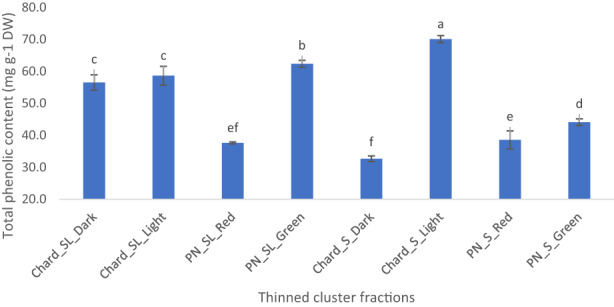
Total phenolic content in thinned cluster fractions. Different letters (a–f) indicate statistical significance (*p* < .05) between different thinned cluster fractions for total phenolic content.

The total phenolic content (TPC) determination was the starting point for the thinned grape cluster characterization because it provided important information, although unspecific, about the relative composition of the thinned grape cluster sample (Fontana et al., 2013). The composition and concentration of phenolics in grapes vary greatly due to variety and degree of ripeness (Rockenbach et al., 2011), and environmental and vineyard management factors such as soil type (Wang et al., 2015) and irrigation strategy (Gamero et al., 2014). Thus, it is not surprising to see a 2.1‐fold and 1.7‐fold variations of TPC in Chardonnay and Pinot noir fractions, respectively. As shown in Figure 1, Chard_S_Light, which was considered the least mature among all fractions, had the highest TPC at 70.1 ± 1.1 mg g^−1^ in dry weight (DW). The highest TPC for Pinot noir was detected in its green seedless fraction at 62.4 ± 1.1 mg g^−1^ DW. Within the Chardonnay fractions, the less mature fractions (Light) had higher TPC compared to the more mature fractions (Dark) in both seed and seedless forms although no significant difference was found in two seedless fractions (*p* > .05). The same trend was also observed in Pinot noir fractions as the less mature green fractions for both seed and seedless had higher TPC compared to the more mature red fractions. Pantelić et al. (2016) also found that TPC values were significantly higher in seeds than in skins and pulps in seven red and six white grapevine varieties grown in Serbia; our Chardonnay Dark and Pinot noir Green fractions were on the opposite where the seedless fractions yielded higher TPC for both varieties. Typically, the amount of TPC in the white grape varieties is lower than that of red grapes due to lack of anthocyanins synthesis (Ivanova et al., 2011), yet, previous studies have found that the TPC of grapes mainly depends on the varietal differences, not on grape skin color (Breksa III et al., 2010; Yang et al., 2009). Furthermore, in this study, thinned clusters for Pinot noir were collected at the beginning of veraison before anthocyanins accumulation reached its peak. Variations among grape fractional sample preparation (freeze‐drying vs. wet samples), TPC extraction (differences in extraction equipment, aqueous/organic solvent type and ratio, time, and temperature), and data reporting format (dry weight vs frozen weight vs. volume) could result in significant TPC differences from study to study for the same varieties (Di Lecce et al., 2014; González et al., 2020; Ivanova et al., 2011; Kwiatkowski et al., 2020; Pantelić et al., 2016; Yang et al., 2009).

Flavanol monomers and procyanidins are abundant in cocoa and grapes. In cocoa, procyanidins are the major flavanols which consist of oligomers as well as polymers of catechins and epicatechins commonly bonded through a C4‐C6 or C4‐C8 linkage (Gu et al., 2006). Grapes are also rich sources for oligomeric and polymeric procyanidins with studies showing oligomeric procyanidins such as dimers (B1‐B4) and trimers (C1) being easier to be absorbed in vivo (Holt et al., 2002; Tsang et al., 2005). Although cocoa‐based products can be perceived as “healthy” among consumers because cocoa flavanols exhibited numerous health‐promoting properties (Al‐Dashti et al., 2018; Arranz et al., 2013; Holt et al., 2006; Martín & Ramos, 2017), the amount of bioactive and bioavailable cocoa flavanols, mainly (−)‐epicatechin, (+)‐catechin, and procyanidin dimers and trimers, vary greatly among cocoa‐based products (Gu et al., 2006) thus are unlikely to achieve the same level of health‐promoting effects in vivo. Therefore, it is crucial to quantify the flavanol monomers and oligomeric procyanidins content in the grape thinned clusters to determine their bioactive potential when compared with cocoa ingredients such as cocoa powder.

**FIGURE 2:**
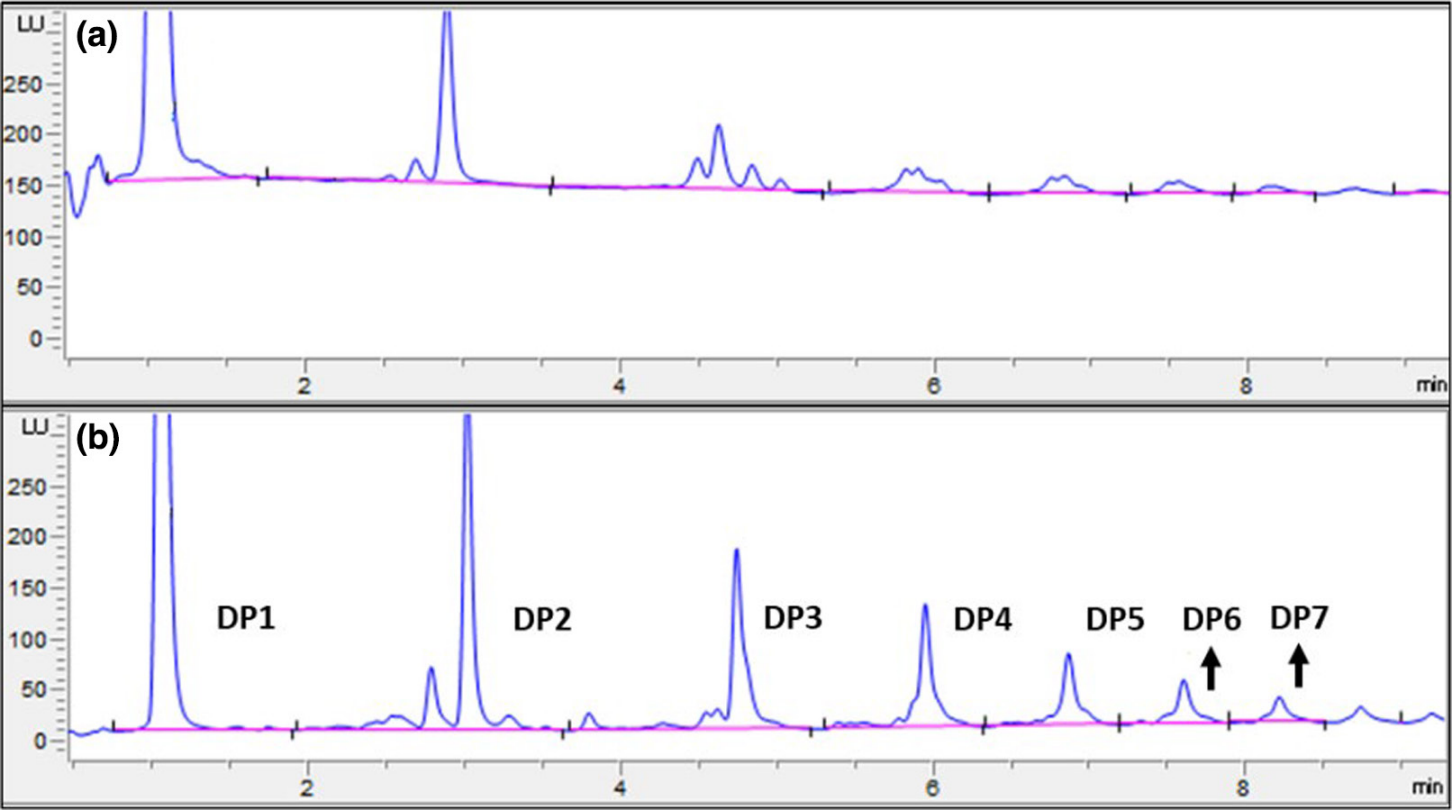
UPLC‐FLD chromatograms of flavanols and procyanidins (DP 1–7) in (a) Pinot noir_Seed_Red (PN_S_Red); (b) Acticoa™ cocoa powder.

**FIGURE 3:**
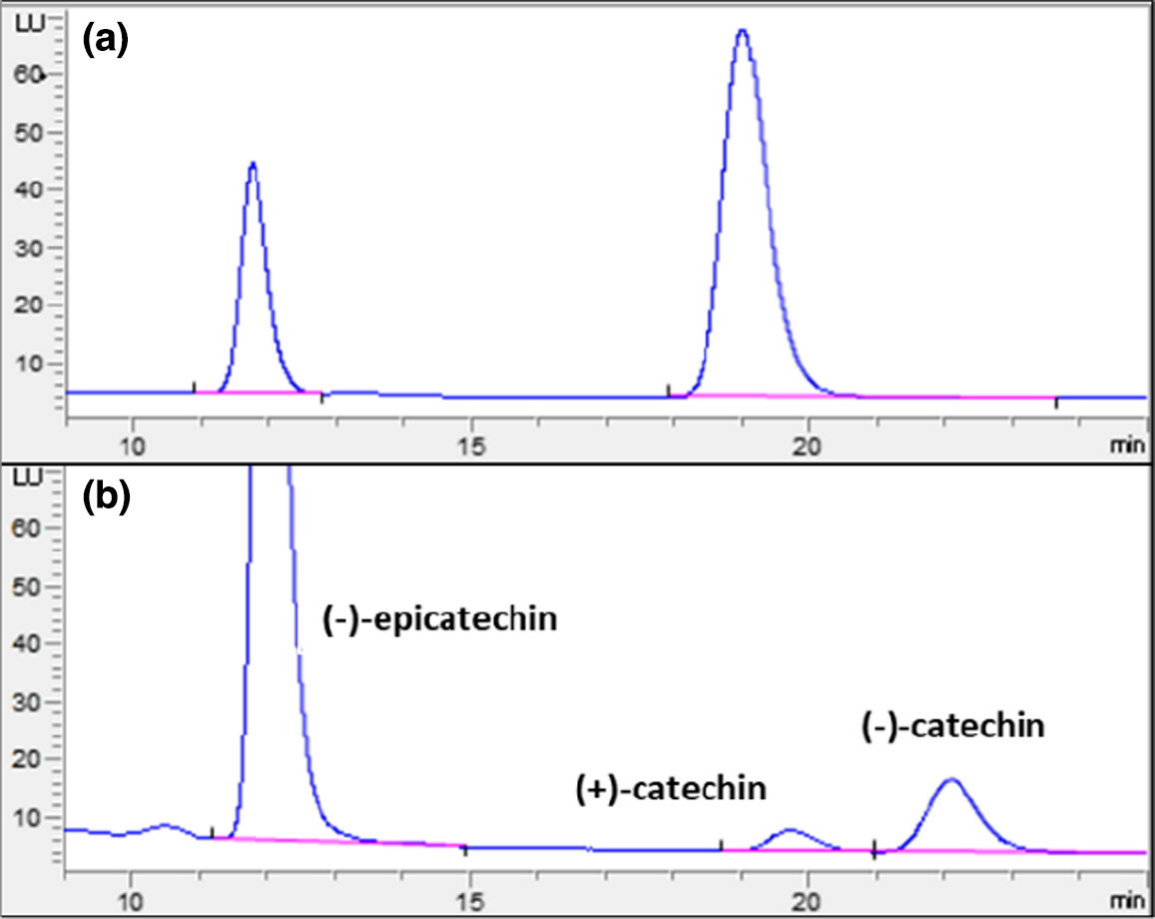
UPLC‐FLD chromatograms of catechin and epicatechin enantiomers in (a) Pinot noir_Seed_Red (PN_S_Red); (b) Acticoa™ cocoa powder.

A new cocoa extract reference NIST RM8403 was used as an external standard in the recently approved 2020.05 AOAC official method which allows more accurate and reliable determination of cocoa flavanols and procyanidins (DP 1–7) using UPLC‐FLD (Bussy, Hewitt, et al., 2021). The current study successfully applied this method to the Chardonnay and Pinot noir thinned cluster fractions as well as two commercial cocoa powders in the context of catechin, epicatechin, and their oligomeric procyanidins (Table 1). An example of chromatogram is shown in Figure 2. Due to concerns of flavanol monomer epimerization during cocoa production which might yield substantially less bioactive (−)‐catechin from naturally occurring (−)‐epicatechin (Keen & Holt, 2012) the specific concentrations of catechin and epicatechin enantiomers of thinned clusters and cocoa powders are also provided in Table 2 using chiral chromatography (Machonis et al., 2014; Figure 3) to facilitate future epidemiological study designs.

**TABLE 1:** Content of flavanols and procyanidins (DP 1–7) in two commercial cocoa powders and eight thinned cluster fractions.

	DP 1	DP 2	DP 3	DP 4	DP 5	DP 6	DP 7	Total DP 1–7
	Concentration in mg g^−1^ DW for thinned clusters and mg g^−1^ whole powder (including fat) for cocoa powder							
Mullica™ cocoa powder	1.23 ± 0.03^g^	0.91 ± 0.04^g^	0.63 ± 0.01^f^	0.31 ± 0.02^g^	0.21 ± 0.03^g^	0.10 ± 0.02^g^	0.0896 ± 0.0006^h^	3.5 ± 0.2^h^
Acticoa™ cocoa powder	15.1 ± 0.1^b^	10.7 ± 0.1^a^	12.3 ± 0.1^a^	12.3 ± 0.1^a^	10.5 ± 0.1^a^	8.8 ± 0.1^a^	6.81 ± 0.09^a^	76.6 ± 0.8^a^
Chard_SL_Dark	12.2 ± 0.6^c^	3.20 ± 0.07^b^	2.67 ± 0.03^c^	2.14 ± 0.06^d^	2.11 ± 0.01^d^	2.03 ± 0.03^cd^	1.54 ± 0.03^cd^	25.9 ± 0.9^c^
Chard_SL_Light	10.6 ± 0.1^d^	3.12 ± 0.02^bc^	2.37 ± 0.04^d^	1.79 ± 0.02^e^	1.81 ± 0.05^e^	1.71 ± 0.02^e^	1.31 ± 0.01^e^	22.8 ± 0.3^de^
PN_SL_Red	5.5 ± 0.1^f^	1.49 ± 0.07^f^	1.62 ± 0.03^e^	1.36 ± 0.01^f^	1.31 ± 0.02^f^	1.13 ± 0.02^f^	0.82 ± 0.02^g^	13.2 ± 0.3^g^
PN_SL_Green	9.4 ± 0.2^e^	2.6 ± 0.4^cd^	3.22 ± 0.05^b^	2.57 ± 0.04^c^	2.39 ± 0.04^c^	2.14 ± 0.03^c^	1.63 ± 0.02^c^	24.0 ± 0.8^cd^
Chard_S_Dark	11.7 ± 0.2^cd^	1.68 ± 0.03^ef^	1.62 ± 0.02^e^	1.4 ± 0.01^f^	1.3 ± 0.05^f^	1.23 ± 0.03^f^	1.003 ± 0.004^f^	19.9 ± 0.3^f^
Chard_S_Light	25.3 ± 0.5^a^	3.49 ± 0.02^b^	3.41 ± 0.03^b^	3.00 ± 0.02^b^	2.98 ± 0.03^b^	2.72 ± 0.03^b^	2.22 ± 0.03^b^	43.1 ± 0.6^b^
PN_S_Red	10.5 ± 0.3^de^	1.81 ± 0.04^ef^	2.22 ± 0.03^d^	1.82 ± 0.03^e^	1.75 ± 0.04^e^	1.55 ± 0.02^e^	1.13 ± 0.03^f^	20.8 ± 0.5^ef^
PN_S_Green	12.37 ± 0.09^c^	2.2 ± 0.1^de^	2.69 ± 0.05^c^	2.26 ± 0.04^d^	2.14 ± 0.04^d^	1.92 ± 0.02^d^	1.49 ± 0.02^d^	25.0 ± 0.4^c^

*Note*: Letters (a–h) indicate statistical significance (*p* < .05) between different samples for each and total procyanidins with different degree of polymerization from 1 to 7.

**TABLE 2:** Content of (−)‐epicatechin, (+)‐catechin, and (−)‐catechin in thinned cluster fractions and cocoa powders.

	(−)‐Epicatechin	(+)‐Catechin	(−)‐Catechin
	Concentration in mg g^−1^ DW for thinned clusters and mg g^−1^ whole powder (including fat) for cocoa powder		
Mullica™ cocoa powder	0.51 ± 0.03^h^	0.017 ± 0.002^h^	0.44 ± 0.02^b^
Acticoa™ cocoa powder	13.4 ± 0.4^a^	0.54 ± 0.02^g^	1.16 ± 0.03^a^
Chard_SL_Dark	5.74 ± 0.02^c^	6.76 ± 0.02^d^	ND
Chard_SL_Light	5.06 ± 0.02^d^	5.38 ± 0.02^e^	ND
PN_SL_Red	1.741 ± 0.004^g^	3.55 ± 0.01^f^	ND
PN_SL_Green	3.51 ± 0.01^e^	5.47 ± 0.02^e^	ND
Chard_S_Dark	4.62 ± 0.02^d^	6.67 ± 0.03^d^	ND
Chard_S_Light	9.88 ± 0.04^b^	12.98 ± 0.04^a^	ND
PN_S_Red	2.33 ± 0.02^f^	7.41 ± 0.04^c^	ND
PN_S_Green	3.89 ± 0.01^e^	7.77 ± 0.03^b^	ND

*Note*: Letters (a–h) indicate statistical significance (*p* < .05) between different samples for each enantiomer.

Table 1 shows that the highest total amount of flavanols and procyanidins (DP 1–7) was detected in Acticoa™ cocoa powder at 76.6 ± 0.8 mg g^−1^ whole powder while the lowest amount was found in Mullica™ cocoa powder at only 3.5 ± 0.2 mg g^−1^ whole powder. Acticoa™ cocoa powder is a functional ingredient in which 80% of the natural flavanol content of raw cocoa is preserved due to gentle cocoa processing and is often used in epidemiological studies (Bisson et al., 2008; Rynarzewski et al., 2019), whereas Mullica™ cocoa powder is a traditionally Dutched cocoa powder that has been widely used in ice cream and chocolate‐flavored beverages. Mullica™ cocoa powder was also significantly lower in each DP compared to thinned clusters. Compared to Mullica™ cocoa powder, thinned clusters yielded 3.8–12.3 times more total flavanols and procyanidins DP 1–7. In particular, the highest flavanols and procyanidins (DP 1–7) content was found in Chard_S_Light at 43.1 ± 0.6 mg g^−1^ DW, while the lowest content was 13.2 ± 0.3 mg g^−1^ DW in PN_SL_Red. Grapes have abundant non‐extractable phenolics, mainly high‐molecular‐weight proanthocyanidins (Serrano et al., 2018), the flavanols and procyanidins (DP 1–7) in Table 1 mostly included extractable small‐molecule monomers, dimers, and trimers which could explain the total content of flavanols and procyanidins (DP 1–7) of thinned clusters followed similar trend compared to their TPC. Both green seedless and seed fractions of Pinot noir had significantly higher flavanols and procyanidins (DP 1–7) compared to the more mature red fractions (24.0 ± 0.8 vs. 13.2 ± 0.3 mg g^−1^ DW for seedless and 25.0 ± 0.4 vs. 20.8 ± 0.5 mg g^−1^ DW for seed). The same trend was observed for Chardonnay fractions as the less mature (Light) seed had the highest flavanols and procyanidins (DP 1–7) compared to its more mature dark seed fraction (43.1 ± 0.6 vs. 19.9 ± 0.3 mg g^−1^ DW). However, for the Chardonnay seedless fractions, the dark fraction had a significantly higher amount than the light fraction (25.9 ± 0.9 vs. 22.8 ± 0.3 mg g^−1^ DW). Ivanova et al. (2011) have found that the total content of catechin, epicatechin, and procyanidin dimers B1‐B4 in Chardonnay and Merlot grown in R. Macedonia were significantly higher in seeds rather than in skins and pulp at veraison. Our data on flavanols and procyanidins DP 1–2 (monomers and dimers) are in accordance with their findings as the highest content was found in Chardonnay seeds (light and dark combined), followed by Pinot noir seeds (green and red combined), Chardonnay seedless (light and dark combined), and Pinot noir seedless (green and red combined). Overall, 2.2‐fold and 1.2‐fold variations of DP 1–2 were found in Chardonnay and Pinot noir thinned cluster fractions, respectively. Previous studies also showed that flavanol monomers in white and red grapes slow down or stop accumulating from veraison to ripening while procyanidin dimers B1‐B4 increase slightly until the intermediate phase (Ivanova et al., 2011; Jordão et al., 2001a, 2001b; Kennedy et al., 2000; Lorrain et al., 2011). These all indicated that grape thinned clusters at veraison could be richer sources of monomeric and oligomeric flavanols and procyanidins compared to grapes during berry formation and berry ripening.

With the increasing interest in utilizing specific bioactive catechin and epicatechin enantiomers, mainly (−)‐epicatechin and (+)‐catechin, in epidemiological studies (Al‐Dashti et al., 2018; Donovan et al., 2006; Keen & Holt, 2012), we separated catechin and epicatechin enantiomers using chiral chromatography and presented the data in Table 2. Like grapes, the predominant forms of flavanol monomers in natural cacao beans are (−)‐epicatechin and (+)‐catechin. However, Payne et al. (2010) have found that various processing techniques used in cocoa production, such as fermentation and roasting at over 70°C, significantly decrease the total catechins amount and generate significant amounts of (−)‐catechin from (−)‐epicatechin epimerization, respectively. The Dutch processing (alkalization) is widely used in cocoa production where the cocoa is treated with an alkali solution (potassium or sodium carbonate) to reduce the acidity and to intensify the chocolaty flavor in the final powder (Beg et al., 2017). Compared to natural cocoa powders, Dutch processing also caused a greater loss in both epicatechin (up to 98%) and catechin (up to 80%) according to Payne et al. (2010) Not surprisingly, Acticoa™ cocoa powder being the functional ingredient used in cocoa‐based products had the highest (−)‐epicatechin at 13.4 ± 0.4 mg g^−1^ whole powder, while thinned cluster fractions had amounts of (−)‐epicatechin in the range of 1.741 ± 0.004 (PN_SL_Red) to 9.88 ± 0.04 mg g^−1^ DW (Chard_S_Light). There were 3.4 and 19.4 times higher than Mullica™ cocoa powder at 0.51 ± 0.03 mg g^−1^ whole powder. Contrary to cocoa powders, Chardonnay and Pinot noir thinned clusters had more (+)‐catechin than (−)‐epicatechin which was consistent with other studies that included these two varieties (Pantelić et al., 2016; Rockenbach et al., 2011). Specifically, PN_SL_Red (lowest in catechin and epicatechin concentrations) and Chard_S_Light (highest in catechin and epicatechin concentrations) had 208.8 and 763.5 times more and 6.6 and 24.0 times more (+)‐catechin than Mullica™ and Acticoa™ cocoa powder, respectively. As (+)‐catechin has been shown to have pharmacological effects including antimicrobial (Takagaki & Nanjo, 2016), antioxidative (Bentz et al., 2017), and had about five times higher bioavailability than (−)‐catechin (Ritter et al., 2010), thinned clusters could complement cocoa ingredients—a small portion of grape thinned cluster as an ingredient would likely make a positive impact on the (+)‐catechin and total catechins content of cocoa‐based products.

## 4 CONCLUSIONS

Consumer interests in the consumption of healthy foods are rising worldwide. Cocoa‐based products are well‐perceived as good sources of dietary flavanols which are health‐promoting and can be a part of healthy diet. In this study, plant‐based natural products thinned cluster seed and seedless fractions of Chardonnay and Pinot noir grown in North Coast California exhibited significant amounts of flavanol monomers (−)‐epicatechin, (+)‐catechin, and their oligomeric procyanidins comparing to Acticoa™ and Mullica™ cocoa powders—two commercial cocoa ingredients that have been widely used in food and beverage industry and even epidemiological studies. Our preliminary finding indicated these underutilized grape thinned clusters at veraison could make good candidates for functional ingredients in cocoa‐based products to further enhance the overall flavanol content. The combination of cocoa and grape thinned clusters enhances the consumer‐appealing idea of “flavanol‐rich” food by enabling the possibility of producing a variety of flavanol‐enriched cocoa‐based food products such as chocolate milk, milk chocolate, chocolate syrup, and hot cocoa mix, which typically contain very little cocoa flavanols (Arts et al., 2000; Risner & Kiser, 2008). With a mission of helping the wine industry to reduce waste during the wine‐making process and identifying new upcycling opportunities, we are continuing this work with chemists, microbiologists, nutrition scientists, sensory scientists, and processors to create and test prototypes of various cocoa‐based products incorporated with a larger selection of thinned cluster ingredients to provide a comprehensive picture of the bioactive flavanols in the products. Ongoing work includes gaining an understanding of how different processing techniques affect the product flavanol profile, consumer acceptance toward the products, and importantly, the mechanism of health‐promoting action of the products in vitro and in vivo.

## AUTHOR CONTRIBUTIONS

Selina C. Wang carried out funding acquisition and project supervision. Xueqi Li was involved in experimental design and data analysis and manuscript writing. Xueqi Li and Selina C. Wang carried out manuscript reviewing and editing.

## CONFLICT OF INTEREST STATEMENT

The authors declare no competing interest.

## ETHICAL APPROVAL

Ethics approval was not required for this research.

## Data Availability

The data that support the findings of this study are available from the corresponding author upon reasonable request.
